# Impact of the Different Preparation Methods to Obtain Autologous Non-Activated Platelet-Rich Plasma (A-PRP) and Activated Platelet-Rich Plasma (AA-PRP) in Plastic Surgery: Wound Healing and Hair Regrowth Evaluation

**DOI:** 10.3390/ijms21020431

**Published:** 2020-01-09

**Authors:** Pietro Gentile, Claudio Calabrese, Barbara De Angelis, Laura Dionisi, Jacopo Pizzicannella, Ashutosh Kothari, Domenico De Fazio, Simone Garcovich

**Affiliations:** 1 Plastic and Reconstructive Surgery, Surgical Science Department, University of Rome “Tor Vergata”, 00179 Rome, Italy; bdeangelisdoc@gmail.com; 2 San Rossore Clinic, Breast Unit Department, 56122 Pisa, Italy; claudiocalabrese.it@gmail.com; 3 Dionisi Law Firm, 00133 Rome, Italy; studio@avvocatolauradionisi.it; 4 Ss. Annunziata Hospital, ASL02 Lanciano-Vasto Chieti, 66100 Chieti, Italy; jacopo.pizzicannella@unich.it; 5 Head of Breast Surgery Unit, Guy’s Hospital, Guy’s and St. Thomas’ NHS Foundation Trust, London SE1 9RT, UK; Ashutosh.Kothari@gstt.nhs.uk; 6 Institute of Plastic Surgery, Galeazzi Hospital, 20122 Milan, Italy; info@domenicodefazio.it; 7 Institute of Dermatology, Fondazione Policlinico Gemelli IRCSS, Università Cattolica del Sacro Cuore, 00168 Rome, Italy; simgarko@yahoo.it

**Keywords:** platelet-rich plasma, PRP, autologous platelet-rich plasma, A-PRP, PRP kit, PRP procedures, activated PRP, non-activated PRP, regenerative plastic surgery

## Abstract

Autologous therapies using platelet-rich plasma (PRP) need meticulous preparation—currently, no standardised preparation technique exists. Processing Quantitative Standards (PQSs) define manufacturing quantitative variables (such as time, volume and pressure). Processing Qualitative Standards (PQLSs) define the quality of the materials and methods of manufacturing. The aim of this review is to use existing PQSs and PQLs to report the in vivo/in vitro results obtained by using different Kits, that utilise different procedures (classified as Closed-Technique and Opened-Technique) to isolate autologous human activated (AA-PRP) or non-activated PRP (A-PRP). PQSs included the volumes of blood collected as well as the reagents used, the time/gravity of centrifugation, and the duration, temperature and tilt level/speed of centrifugation. PQLSs included the use of Calcium Chloride CaCl_2,_ Kit weight, transparency of Kit components, the maintenance of a closed sterile processing environment and the use of a small centrifuge. Eight CE marked devices for PRP extraction were evaluated: Angel^®^, Biomed^®^, Cascade^®^ and Selphyl^®^, Mag-18^®^, i-Stem^®^, MyCells^®^ and Regenlab^®^. Using a Kit with the PQSs and PQLSs described in this study enables the isolation of A-PRP, thereby meeting consensus quality criteria. As our understanding of Critical Quality Attributes (CQAs) of A-PRP continues to evolve, especially with respect to purity and potency, adjustments to these benchmark PQSs and PQLs will hopefully help isolate A-PRP of desired CQAs with greater reproducibility, quality, and safety. Confirmatory studies will no doubt need to be completed.

## 1. Introduction

A scientific need exists for the development of biotechnologies to improve regenerative strategies in plastic surgery, and in particular, hair loss. In order to achieve this, advances in tissue engineering (TE) should revolve around the development of new autologous-technologies. In this case, autologous non-activated Platelet-rich plasma (A-PRP) or activated Platelet-rich plasma (AA-PRP) to improve hair re-growth and wound healing by in vitro and in vivo regeneration and biostimulation.

The use of autologous platelets derived growth factors (GFs), contained in A-PRP and/or AA-PRP, may find support in TE through their activity to stimulate cell differentiation, proliferation and neo-angiogenesis, thereby aiding the wound healing process [1,2,3] and hair regrowth [4]. In fact, A-PRP and AA-PRP contain hundreds of signalling proteins and GFs such as basic fibroblast growth factor (bFGF), platelet-derived growth factor (PDGF), vascular endothelial growth factor (VEGF), epidermal growth factor (EGF), transforming growth factor-β (TGF-β) and insulin-like growth factor-1 (IGF-1) that are discharged after platelet activation [4]. Each one of these major GFs is involved in a specific biomolecular activity during hair re-growth and wound healing. The Platelet Physiology Subcommittee of the Scientific and Standardization Committee (SSC) of the ISTH formed a working party of experts with the aim of producing consensus recommendations for guidance on the use of platelets in regenerative medicine [5]. The RAND method is used, which obtains a formal consensus among experts particularly when scientific evidence is absent, scarce and/or heterogeneous. Using this approach, each expert scored as ‘appropriate’, ‘uncertain’ or ‘inappropriate’ a series of 45 statements about the practice of regenerative medicine with platelets, which included different sections on general aspects, platelet preparations, clinical trial design, and potential utility in different clinical scenarios [5].

In fact, there are a considerable number of papers published on A-PRP and/or AA-PRP, but the results are often contradictory. It is possible to identify different types of PRP preparations by way of their cell content and fibrin architecture as follows:

1. Leukocyte-poor PRP (LP-PRP). Suspension without leukocytes and with a low-density fibrin network after activation;
2. Leukocyte-rich PRP (LR-PRP). Suspension with leukocytes and a low-density fibrin network after activation;
3. Leukocyte-poor platelet-rich fibrin (LP-PRF). Suspension without leukocytes and a high-density fibrin network.
4. Leukocytes-rich platelet rich fibrin (LR-PRF). Suspension with leukocytes and a high-density fibrin network.

As previously highlighted, there are several existing procedures for the preparation of A-PRP and/or AA-PRP depending on the different centrifugation time and right per minutes (RPM) used, platelets number and GFs and chemokines availability. There is also a wide biological (between patients) and temporal (day to day) variation [6]. As such, it is difficult to assess which kit for PRP preparation is better and which is deficient [7]; different PRP products could be more, or then less effective in the treatment of different types of tissues and pathologies. The impact and efficacy of PRP in general remains contested, and a standardized procedure has not yet been established [8]. Therefore, appropriate PRP preparations should be select after carefully considering their biomolecular specs and intended indications for use in patients [9].

This manuscript aims to inform about the impact of different preparation methods to obtain A-PRP and/or AA-PRP on wound healing and hair re-growth. The characteristics of the devices analysed are reported, intending to clarify any doubts regarding the classification of devices used in closed or open techniques.

This review is performed following the principles outlined in the Declaration of Helsinki and internationally consented ethics in clinical research [10]. A quality assessment is carried out based on the Strengthening the Reporting of Observational Studies in Epidemiology (STROBE) checklist [11].

## 2. Methods

### 2.1. European and Italian Rules

Human autologous PRP must be considered an emocomponent. In order to better understand the context of current European Rules, it is necessary to differentiate between emocomponents for topical use and those used in cell therapy, that involve complex bioprocessing techniques of the therapeutic cells. All rules have a common purpose: ensure voluntary donation of emocomponents; self-sufficiency of blood, emocomponents and plasma-derived products; guarantee the donors and recipients health; quality and safety of the procedures and the products of transfusion medicine. The European Rules related to the use of PRP have been represented by Decree of 9 November 2007, n. 207, ‘Implementation of Decree 2005/61/EC implementing Directive 2002/98/EC for what concerns the prescription in means of traceability of blood and blood components intended for transfusion and the notification of adverse and severe reactions’ and the Legislative Decree of 9 November 2007, n. 208, ‘Implementation of Directive 2005/62/EC implementing Directive 2002/98/EC with regard to standards and specifications relating to a quality system of blood’.

Currently, especially in Italy, preparation of PRP must be performed as per, “Decree of the Blood, 2 November 2015”, which details dispositions related to quality and safety parameters of blood and emocomponents, in which all patients must receive detailed oral and written information about the study, including the risks, benefits and alternative therapies, and sign an informed consent form before any study procedures, according to transfusional service.

This review has been the subject of a research contract (D.R. 1467/2017) and a university master’s degree called “Regenerative surgery and medicine in wound care management”, of “Tor Vergata” University, Rome, Italy.

### 2.2. Closed and Opened Technique to Obtain PRP

The classification of the devices is based on the characteristics related to the procedure to obtain A-PRP and AA/PRP suspensions, identifying “Closed” and “Opened” techniques.

“Closed technique”: Involves the use of commercial devices that have CE marking (including centrifugation and application equipment) in which the product must not be exposed to the environment.

“Open technique”: Is a procedure in which the product is exposed to the environment within the working area and it enters in contact with different materials and equipment necessary for obtaining and deploying it, such as pipettes or tubes for product collection. During the processing of blood to obtain PRP through the open technique one must ensure that the product is not contaminated with microbes during handling.

### 2.3. Processing Quantitative Standards (PQSs) and Processing Qualitative Standards (PQLSs)

Currently, there is a distinct lack of a standardised preparation technique. Processing Quantitative Standards (PQSs) define manufacturing quantitative variables (such as time, volume and pressure). Processing Qualitative Standards (PQLSs) define the quality of the materials and methods of manufacturing. The objective of this study is to describe the in vivo/in vitro results obtained by different Kits and with different procedures (closed vs. opened) to isolate human PRP using PQSs and PQLSs. PQSs included the volumes of blood harvested and reagents used, the duration/gravity of centrifugation, and the time, temperature and tilt level/speed of incubation and/or centrifugation. PQLSs included the use of CaCl_2,_ a processing time of 30 min, kit weight, transparency of Kit components, the maintenance of a closed sterile processing environment and the use of a small centrifuge and incubating rocker. Using the Kit with the PQSs and PQLSs described in this study enables the isolation of PRP, which meets consensus quality criteria. As the discoveries of new Critical Quality Attributes (CQAs) of PRP continue to be made, for example with respect to purity and potency, adjustments to these benchmark PQSs and PQLs will hopefully isolate PRP of various CQAs with greater reproducibility, quality, and safety. Confirmatory studies will no doubt need to be completed.

### 2.4. A-PRP and AA-PRP Devices

Eight different devices are analyzed and reviewed. Currently, each of them could have several iterations that refer to commercial variants of a combination of fungibles but that have a common denominator which is the device described. The devices used, listed alphabetically by trade name are as follows: Angel^®^ (Arthrex, Inc. Corporate Naples, FL, USA, 1370), Cascade^®^ (Musculoskeletal Transplant Foundation, Edison, NJ, USA, 08837) and Selphyl^®^ (Factor Medical LLC, Langhorne, PA, USA, 19047), C-Punt^®^ (Biomed Device, Modena MO, Italy, 41126), i-Stem^®^ Preparation System (i-Stem, Biostems, Co., LTD., Seoul, Korea, 138-843), MAG-18^®^ (DTS MG Co., Ltd., Seoul, Korea, #B108-147), MyCells^®^ (Kaylight Technologies Ltd., Holon, Israel), Regenlab^®^ (En Budron b2, 1052 Le Mont-sur-Lausanne, Swiss).

The Angel^®^ device uses software and programmed centrifugations to obtain A-PRP. The device requires experience on the part of the user and allows the user to select a wide range of platelet concentrations (from 3× to 18×). In this system, the device acts as a fractionator; the Plasma is the centrifuge itself with a complex expendable set. The content is not exposed to the environment and it follows that this represents a ‘closed technique’. The device is used to prepare A-PRP (3 mL) starting from 120 mL of whole blood. The A-PRP is then combined with 5 mL of platelet-poor plasma to produce 8 mL of A-PRP with a 5-fold increase in platelet concentration over whole blood. A-PRP collected is then activated through the addition of 10% (*v*/*v*) calcium gluconate to obtain AA-PRP [4]. 

Using the Cascade^®^ or Selphyl^®^ device, AA-PRP is prepared from a small volume of blood (18 mL) collected in two different tubes (9mL in each one—yellow in colour) from a peripheral vein using sodium citrate as an anticoagulant (ACD). The tubes are centrifuged at 1100 *g* for 10 min (min) with the aim of obtaining a platelet pellet. Later, the suspension contained in the tubes is activated by switching into two tubes (red colour) containing CaCl_2_ to induce platelet activation and exocytosis of the alpha granules [3].

C-Punt^®^ consisting of a 60 mL syringe in which whole blood (55 mL) is collected from a peripheral vein using ACD. The syringe is centrifuged at 1200 rpm for 10 min; after this, the 23 mL of autologous platelet suspension obtained—(Platelet-Poor Plasma—PPP and Platelet-rich plasma—PRP), is inserted in a platelet selector device and at the end of the procedure, 9 mL of A-PRP are harvested [4].

Using an hourglass system, the i-Stem^®^ Preparation System, blood (17.7 mL) is drawn and ACD (2.2 mL) is used as an anticoagulant. After the first spin (centrifugation at 3000 RPM for 6 min), the PPP portion (1 mL) and RBC (Red blood cells) (2 mL) is removed and the suspension obtained is centrifuged for a second time (3000 rpm for 3 min). At the end of the procedure, 15 mL of A-PRP are obtained.

Mag-18 PRP^®^ is a hourglass system in which 18mL of whole blood and 1mL of ACD are collected and centrifuged twice—the first time, at 3000 RPM for 6 min and a second time at 3400 RPM for 2 min. 1.5 mL of A-PRP is obtained in the middle portion of hourglass, indicated by a buffy coat.

MyCells^®^ A-PRP preparation involved centrifuging a patient’s blood sample (10 mL). A vial of blood is centrifuged for 10 min at 3000 RPM [12].

PRP Regen Blood Cell Therapy^®^ tubes are used to prepare A-PRP (15 mL, 5 mL per BCT tube) from whole blood (24 mL) taken from a peripheral vein using ACD. The top 2 mL of A-PRP from each tube is then discarded, giving 9 mL of A-PRP with a five-fold increase in platelet concentration over whole blood. Alternatively, a self-contained disposable kit (RegenKit^®^; RegenLab^®^) is used to process 40 mL of venous peripheral blood. Blood was collected in five ATS (autologous thrombin serum) Regen tubes (8 mL each). All tubes are centrifuged at 1500 *g* for 15 min at room temperature using the universal centrifuge Regen Lab PRP-Centri (Regen Lab SA, Le Mont-sur-Lausanne, Switzerland).

After centrifugation, PRP activated (AA-PRP) by autologous thrombin becomes consolidated in the tube [2,4].

Details of all products tested are reported in Table 1.

## 3. Discussion

The high price differentials between devices with similar yields and seemingly similar procedures and a lack of studies that evaluate and compare the final product present a challenge for physicians seeking to select an appropriate method for PRP preparation. The results analyzed indicate that comparative studies between different systems of PRP preparation may reveal differences in term of wound healing [1,2,3] or hair regrowth [4]. A different impact of A-PRP and/or AA-PRP has been reported, depending on the tissue and pathology being treated.

With regards to hair re-growth, different hair count and hair density results have been reported [4]. In vitro, antiapoptotic effects of A-PRP and AA-PRP have been identified as one of the major contributing factors stimulating hair growth through the activation of the Bcl-2 protein (antiapoptotic regulator) and Akt signalling, prolonging the survival of dermal papilla cells (DPCs) during the hair cycle. In particular, the up-regulation of fibroblast growth factor-7 (FGF-7)/b-catenin signalling pathways with A-PRP treatment is suggested to stimulate hair growth by inducing Human Follicle Stem Cells differentiation as well as prolonging the anagen phase of the hair growth cycle [4]. It also appears to increase the perifollicular vascular plexus through the increase of vascular endothelial growth factor (VEGF) and platelet-derived growth factor (PDGF) levels, which have angiogenic potential [4].

To better explain the different in vivo results obtained using A-PRP and AA-PRP, it appears necessary to report the most recent outcomes in hair density and hair count obtained for these treatments and to compare the results with Human Follicle Stem Cells injection. In detail, 12 weeks after the last injection (A-PRP and AA-PRP have been performed every 30 days, three times), hair density measurements for patients treated with A-PRP and AA-PRP were 65 ± 5 and 28 ± 4 hairs/cm^2^ respectively. The results obtained constitute a 31 ± 2% increase in hair density when A-PRP treatment is performed versus 19% ± 3% increase in hair density when AA-PRP treatment is performed, with a statistically significant difference in hair growth (*p* = 0.0029) [4]. Differences between the 12 weeks follow-up counts and the baseline count for these hair growth parameters were higher in the A-PRP treatment population than in the AA-PRP treatment population as reported in the previous trials performed by Gentile et al. [4]. Twenty-three weeks after the last injection, hair density measurements for patients treated with A-PRP and AA-PRP were 28 ± 2 and 15 ± 3 hairs/cm^2^ respectively [4].

The increase of hair density and hair count parameters for A-PRP over AA-PRP may reflect the greater efficiency of in vivo thrombin to activate platelets and of the body to distribute the contents of activated platelets compared with in vitro calcium activation and injection. Moreover, delivery of A-PRP may enable the production of thromboxane A2 (TXA2) by the platelets once they are activated in vivo, which would, in turn, activate additional platelets and amplify platelet aggregation [13].

Both patient populations treated with A-PRP and AA-PRP respectively showed an improvement in the number of follicular bulge cells and follicles, epidermal thickening, improved vascularization, and a higher number of Ki67+ basal keratinocytes in PRP-treated scalp tissue compared with placebo (saline) [4]. Indeed, histological examination of A-PRP and AA-PRP treated scalp tissue from the authors’ previous work [4] provides such in vivo evidence.

The results obtained with A-PRP and AA-PRP injection may be considered similar to Human Follicle Stem Cells procedure. A recent study by Gentile et al. [14], showed hair density improvement for Human Follicle Stem Cells treatment 23 weeks after the second infiltration (one infiltration was performed every 60 days for two injection cycles) was 29 ± 5% hairs/cm^2^ compared with 28 ± 2 hairs/cm^2^ when A-PRP was used. In fact, Gentile et al. [14] reported, for the first time in literature (2017), preliminary work in which autologous Human Follicle Stem Cells suspension, obtained by mechanical centrifugation of a 2 mm punch biopsy of the scalp, was injected in patients affected by androgenetic alopecia (AGA). In 2019, using the “Gentile protocol” [15] based on mechanical and controlled injections of autologous micrografts containing Human Intra and Extra Dermal Adipose Tissue-Derived Hair Follicle Stem Cells obtained by fragmentation, disaggregation and centrifugation of 2 mm scalp’s punch biopsy, without any commercial kit or device, a 33% ± 7.5% increase of HD increments, after 23 weeks, was reported [15].

As briefly alluded to previously, GFs contained in PRP, in different concentrations depending on the procedure used (A-PRP or AA-PRP), may stimulate both hair growth and tissue regeneration, positively influencing the healing process. 

Each one of GFs is involved in a specific biomolecular pathway during hair regrowth and wound healing. In hair regrowth, evaluated in vitro, EGF stimulates migration and growth of follicle ORS cells by activation of Wnt/β-catenin signalling; b-FGF stimulates hairs’ follicles development; VEGF stimulates peri-follicular vascularization; TGF-β improves the signalling pathways that regulate hair cycle; IGF-1 stimulates migration, proliferation and survival of hair follicle cells; IL-6 is involved in WIHN through STAT3 activation; IGFBP-1 to -6 regulates IGF-1 effects and its interaction with extracellular matrix (ECM) proteins at the hair follicle level; PDGF and PDGFR-β/-α64 up-regulate the genes involved in hair follicle differentiation; PDGF and its receptors are essential for follicular development; Wnt3a is involved in hair follicle development through β-catenin signalling; PGE2 stimulates anagen in hair follicles; PGF2α and analogs stimulate the transition from telogen to anagen; BIO (GSK-3 inhibitor); PGE2 or inhibition of PGD2 or PGD2 receptor D2/ GPR4477 stimulates follicle regeneration; BMP maintains DPC phenotype (crucial for stimulation of hair follicle stem cell); BMPR1a maintains the proper identity of DPCs (essential for specific DPC function); M-CSF and M-CSFR are involved in wound-induced hair re-growth [10].

In wound healing, GFs may reduce bleeding and can accelerate healing time [12]. In vitro, the EGF, TGF-beta and FGF family, VEGF, granulocyte-macrophage colony-stimulating factor (GM-CSF), PDGF, connective tissue growth factor (CTGF), interleukin (IL) and the tumour necrosis factor-alpha (TNF-alpha) family can stimulate neoangiogenesis, cell recruitment, growth and morphogenesis, constituting a three-dimensional matrix that allows cellular arrangement into a correct three-dimensional organization [12,16]. For this reason, PRP may be considered a possible biostimulator. Therefore, PRP’s activation stimulates degranulation where the secretory proteins changed to a bioactive state [17,18]. Active proteins may be secreted, binding to transmembrane receptors of target cells, which include mesenchymal stem cells, osteoblasts, fibroblasts, endothelial cells, and epidermal cells. These agonists bind transmembrane receptors, inducing tissue repair and tissue regeneration [19,20,21].

Another mechanism that induces wound healing is the acceleration of hyaluronic acid (HA) production [12]. HA is a biopolymer identified in the ECM of skin, cartilage, bone and brain, among other tissues [22]. It can hydrate and modulate the cellular microenvironment, while its cell surface receptor bindings induce cell-to-cell adhesions, cell-substrate adhesions, proliferation, and cell migrations. Therefore, HA, as a scaffold, not only facilitates the entry of many cells to the wound site but also contributes to the orientation of the ECM [23] and fibrous component [24]. For this reason, HA may act as a scaffold for PRP, improving healing, accelerating remodelling, cell renovation and restoring function.

Combined treatment in chronic ulcers with PRP as a bio-stimulator and HA as a scaffold engaged in bio-functionalized scaffold could be considered as “gold standard” in vivo practice, especially where autograft or allograft tissue might not be available in sufficient quantity for reconstruction [12]. Several studies displaying the combined use of PRP and HA in wound healing have been reported [2,12].

The authors’ goals are to elaborate a cellular mechanism approach in order to regenerate and promote one’s own natural GFs release.

In vivo, as reported by De Angelis et al. [12] after 30 days, the patients affected by chronic ulcers who underwent combined treatment (A-PRP + HA) had 96.8% ± 1.5% of reepithelization compared to 78.4% ± 4.4% in patients (treated with HA alone; *p* < 0.01). No local recurrence has been observed during the follow-up period [12].

The use of autologous A-PRP/AA-PRP in cell-based therapy and biomaterials, contributes to the regeneration of damaged tissues through their use in isolated suspensions or in combination. 

When informing the choice of PRP device, it is necessary to consider all the information available and not rely purely on the information and data provided by the manufacturer. One must consider requesting the manufacturers to provide external validation or scientific papers that substantiate the data, platelet counts obtained and clinical outcomes in different fields. This information may clarify issues such as performance, concentration or quality of the final product obtained with the devices that, after all, are intended to be used in a patient with a therapeutic purpose. To this purpose, one must question, whether we could justify using a low-price A-PRP and/or AA-PRP kit whose technique, composition, platelet concentration and quality have been not been verified and certified? Absolutely not, and it is for this reason that we should consider using only those products that have the technology to preserve the patient’s blood within a closed system.

In each case, without a standardized method for isolating A-PRP/AA-PRP with a specific CQA, regenerative therapies would be difficult and potentially unreliable.

Single-use kits, containing the components necessary to conduct a specific protocol based on “closed technique”, may be best able to provide such standardization. The components of such kits, with imposed PQSs and PQLSs, may improve the reproducibility of A-PRP/AA-PRP isolation as well as make possible the standardization of processing biological materials. 

## 4. Conclusions

Using the Kit with the PQSs and PQLSs as described in this study enables the isolation of A-PRP/AA-PRP, which meet a consensus quality criteria. As the discovery of new CQAs of PRP evolves, such as with respect to purity and potency, adjustments to these benchmark PQSs and PQLs will hopefully isolate A-PRP/AA-PRP of various CQAs with greater reproducibility, quality, and safety. Confirmatory studies will need to be completed to validate these findings. 

## Figures and Tables

**Table 1:** PRP kits and device tested. Abbreviations: leucocyte platelet-rich fibrin (L-PRF); leucocyte platelet-rich plasma (L-PRP); pure platelet-rich fibrin (P-PRF); Pure platelet-rich plasma (P-PRP).

Type	Activation	System	Device	Manufacturer	Fields	Centrifugation	Ref
L-PRP	A-PRP	Closed	MyCells^®^	(Kaylight Technologies Ltd., Holon, Israel)	Wound healing	2054 *g* × 7 min	[12]
L-PRP	A-PRP/AA-PRP	Closed	Regenkit^®^	(RegenLab En Budron b2, 1052 Le Mont-sur-Lausanne, Swiss)	Wound healing; Hair regrowth	1500 *g* × 15 min	[2,4]
L-PRP	A-PRP	Closed	i-Stem^®^	(i-Stem, Biostems, Co., LTD., Seoul, Korea, 138-843)	Hair regrowth	1008 *g* × 10 min (1st) 1295 *g* × 10 min (2nd)	--
L-PRP	A-PRP	Closed	Mag-18 PRP^®^	(DTS MG Co., Ltd., Seoul, Korea, #B108-147)	Hair regrowth	1008 *g* × 10 min (1st) 1295 *g* × 10 min (2nd)	--
L-PRP	A-PRP	Closed	C-Punt^®^	(Biomed Device, MO, Italy, 41126)	Hair regrowth	161 *g* × 10 min (1st) 287 *g* × 15 min (2nd)	[4]
P-PRP	A-PRP/AA-PRP	Closed	Angel^®^	(Arthrex, Inc. Corporate Naples, FL, 1370)	Hair regrowth	1008 *g* × 15 min	[4]
P-PRP	A-PRP/AA-PRP	Closed	Selphyl^®^	(Factor Medical LLC, Langhorne, PA, 19047)	Wound healing; Hair regrowth; Breast and face when mixed with Fat graft	1100 *g* × 10 min	[3]
P-PRF	A-PRP/AA-PRP	Closed	Cascade^®^	(Musculoskeletal Transplant Foundation, Edison, NJ, 08837)	Wound healing; Hair regrowth; Breast and face when mixed with Fat graft	1100 *g* × 10 min (1st) 1372 *g* × 15 min (2nd)	[3]
